# Upcycling of Expanded Polystyrene Waste-Impregnated PVP Using Wet-Phase Inversion for Effective Microalgae Harvesting

**DOI:** 10.3390/polym16192703

**Published:** 2024-09-25

**Authors:** Tutik Sriani, Muslim Mahardika, Shofa Aulia Aldhama, Chandrawati Putri Wulandari, Gunawan Setia Prihandana

**Affiliations:** 1 Department of Research and Development, P.T. Global Meditek Utama-IITOYA, Sardonoharjo, Ngaglik, Sleman, Yogyakarta 55581, Indonesia; tsriani@iitoya.com; 2 Department of Mechanical and Industrial Engineering, Faculty of Engineering, Universitas Gadjah Mada, Jalan Grafika No. 2, Yogyakarta 55281, Indonesia; muslim_mahardika@ugm.ac.id; 3 Department of Industrial Engineering, Faculty of Advanced Technology and Multidiscipline, Universitas Airlangga, Jl. Dr. Ir. H. Soekarno, Surabaya 60115, Indonesia; aldhama.sa@ftmm.unair.ac.id (S.A.A.); chandrawati.p.w@ftmm.unair.ac.id (C.P.W.)

**Keywords:** expanded polystyrene, polyvinylpyrrolidone, microalgae harvesting, Styrofoam, good health, upcycle

## Abstract

The aim of this study was to investigate the potential of upcycling Expanded Polystyrene (EPS) waste collected from food packaging into a membrane for microalgae harvesting, in which membrane filtration often challenges fouling and pore blocking. The target species is *Spirulina platensis*, with *Chlorella vulgaris* as a comparison agent. The membrane was fabricated from used Styrofoam, which typically ends up as single-use food packaging waste. In this study, PVP was used as an additive at varying concentrations, from 2 wt.% to 8 wt.%. The experimental results indicated that despite varying PVP concentrations, all EPS waste membranes exhibited near-complete recovery of *Spirulina platensis* biomass extraction. Despite the similar harvesting efficiency, EPS/PVP-8 exhibited the largest flux of 970.5 LMH/Bar, which is twice the value of the pristine EPS waste membrane. All membranes were hydrophilic; however, hydrophobicity increased with PVP concentration. SEM micrographs revealed that PVP enlarged the membrane surface pores and improved connectivity within the membrane’s structure, ensuring efficient flow. The EPS waste membrane offers promising insights for sustainable materials and wastewater treatment. The upcycling of EPS waste into flat sheet membranes not only addresses the problem of Styrofoam waste accumulation but also paves the way to transform waste into valuable products.

## 1. Introduction

Expanded Polystyrene (EPS), commonly known as Styrofoam, is a white foam plastic material produced from solid beads of polystyrene. The manufacturing of EPS involves impregnating polystyrene beads with pentane as a foaming agent, allowing 20–50 times volume expansion [1]. Some of EPS’s highlights are its exceptional lightweight and thermal insulation properties. It also offers an economical solution for packaging and construction needs [2]. The white color of EPS imparts a sense of cleanliness and hygiene; thus, it is opted for as a packaging material in food industries and as a takeaway by street food vendors. One important drawback is its lightweight feature makes its bulk volume uneconomical to transport to recycling facilities [3]. Furthermore, in Indonesia, the recycling of EPS faces challenges due to limited techniques and infrastructures. Most recycling centers and local waste banks do not accept EPS waste for processing, thus directing the waste to landfills as residual waste or even contaminating waterways [4]. Given that global polystyrene production is on the rise, finding effective ways to recycle/upcycle polystyrene is the best option to reduce the massive amount of Styrofoam waste.

Some researchers attempted to upcycle Styrofoam waste into high-end products such as air filter media [5], oil–water separators [6], concrete composites [7], microfibers [8], and chemical absorbents [9,10]. In membrane technology, Ghaly et al. [11] investigated two types of high-impact polystyrene (HIPS) to create flat sheet membranes. They revealed that as the concentration of HIPS increased, the membrane’s surface porosity and pore size decreased. Munir et al [12] transformed Styrofoam into a face mask filter using a needleless electrospinning method. It has a hydrophobic surface making it suitable as a good face mask material. The electrospinning method was also used to prepare a Styrofoam-based membrane for desalination purposes [13]. They added zeolite into the process and confirmed that the addition of 30 wt.% zeolites led to an 82.63% decrease in water conductivity.

There has been a growing interest in exploring microalgae to tackle global food insecurity as well as to sustain the environment. One of these microalgae is *Spirulina*, a relatively large microalgae with exceptional nutritional value. For mass production, large-scale *Spirulina* farming is carried out in pools or ponds, which requires efficient harvesting. Membrane filtration of microalgae enables nearly complete biomass recovery and the absence of secondary contamination, while also serving as a water conservation strategy [14]. Nevertheless, membrane filtration is susceptible to fouling and pore blocking [15]. The costs associated with membrane replacement and pumping can also be quite high [16]. In regions of developing countries where the climate is conducive to microalgae farming, membrane technology is still perceived as costly. As a result, using membrane filtration for microalgae harvesting is a challenging endeavor. In their study, Yeo et al. observed that the presence of PVP in the membrane dope solution enhanced phase separation and enlarged the macrovoids within the resulting membranes [17]. Polyvinylpyrrolidone (PVP) has found extensive application as an additive in the fabrication of ultrafiltration membranes, mostly as a pore-forming agent to increase flux [18].

This research explores the utilization of EPS waste as the primary component in a polymeric membrane, aiming to explore its effectiveness in harvesting microalgae. Given that EPS waste is abundantly available and free of charge, the resulting membrane is expected to be cost-effective while also contributing to reducing microplastic reduction in the environment. In this work, PVP was introduced as an additive to assess its impact on membrane performance in rejecting *Spirulina platensis*. The membrane’s morphology was analyzed using scanning electron microscopy (SEM). Furthermore, the EPS waste membrane’s performance was examined via pure water flux, water contact angle, and microalgae harvesting efficiency.

## 2. Materials and Methods

### 2.1. Materials

The EPS waste collected from food packaging was rinsed with pure water to ensure no dust or mechanical impurities were present. The cleaned EPS waste was then compacted to remove air, shredded into tiny pieces, and dried at room temperature. The solvent NMethyl-2-Pyrrolidone (NMP) and the additive Polyvinylpyrrolidone (PVP) were sourced from Merck & Co., Inc., Kenilworth, NJ, USA. The strains of *Spirulina* sp. and *Chlorella* sp. used in this study were sourced from the Center of Excellence for Microalgae Biorefinery at Universitas Gadjah Mada. These microalgae were cultured in saline water and harvested on the 10th day, when they reached their peak density of 95,000 cells/mL and had a protein content exceeding 60% [19].

### 2.2. Synthesis of EPS Waste Solution

In five bottle flasks, the shredded EPS waste and NMP were added to make a 20 wt.% solution. The flasks were heated at 80 °C until the shredded Styrofoam was dissolved completely. Subsequently, PVP, as the additive, was added to each bottle to make five solutions with concentrations of 0, 2, 4, 6, and 8 wt.%. To ensure uniform distribution of PVP in the solution, all bottle flasks were heated and stirred for three hours to generate a homogeneous EPS waste dope solution. After the homogeneous state was reached, all bottle flasks were naturally cooled down at room temperature. The dope solutions were marked as PVP-0, PVP-2, PVP-4, PVP-6, and PVP-8, respectively.

### 2.3. Fabrication of EPS Waste Membrane

**Figure 1:**
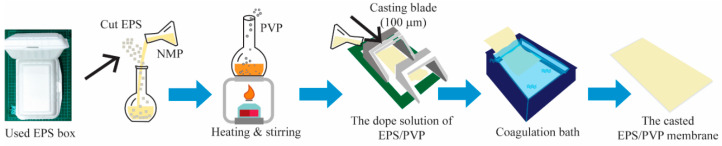
Simplified flow diagram of EPS waste membrane preparation.

A wet-phase inversion method was used to fabricate the EPS waste membrane. Once the dope solution had cooled down, it was carefully poured onto a glass plate. A membrane thickness of 100 µm was achieved by spreading the solution evenly using a casting blade (Elcometer, Manchester, UK). The glass plate was then subsequently transferred to a pure water bath to commence the coagulation of the EPS waste membrane. The membrane was submerged in pure water for a duration of 24 h prior to use. Figure 1 illustrates the preparation and fabrication processes of the EPS waste membrane in this study.

### 2.4. Membrane Characterization

**Figure 2:**
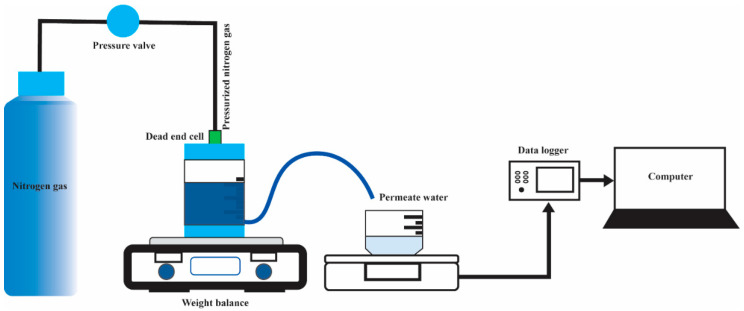
Experimental setup of the water flux test using a dead-end cell unit.

The water flux experiment was conducted for 30 min to achieve stable flux. A dead-end cell (HP4750 Stirred Cell, Sterlitech Corp., Kent, WA, USA) was used to conduct the water flux test, as depicted in Figure 2. The effective membrane area used was 14.6 cm^2^. During the experiment, a fixed pressure of 0.5 bar of nitrogen gas was introduced into the dead-end cell unit to maintain sufficient pressure in the pure water chamber. Throughout the process, the permeate water that passed through the tested membrane was carefully monitored and recorded using a Weighing Environment Logger (AD-1687, A&D, RoHS, Tokyo, Japan). The following equations were used to calculate the volumetric flux ($J_{v}$) and permeability flux ($L_{p}$):

(1) $$J_{v}=\frac{Q}{A\times ∆t}$$

(2) $$L_{p}=\frac{J_{v}}{∆P}$$

where Q is the quantity of the permeate water (in L) during the sampling time, ∆t is the sampling time (in h), A is the area of the membrane ($in\;m^{2}$), and ∆P is the pressure difference (in bar).

In order to evaluate membrane surface hydrophilicity, a 5 µL drop of pure water was placed onto the surface of the dried membrane using a micropipette. A contact angle image of the dropped water was captured using a digital microscope (Dinolite Edge 3.0 AM73915MZTL, AnMo Electronics, New Taipei City, Taiwan). The water droplet angle was determined using AUTOCAD (v.2022). To ensure accuracy, the contact angles for each membrane were measured three times and the average values were calculated. The membrane surfaces and cross-sectional morphologies were analyzed using scanning electron microscopy (SEM Phenom ProX, Thermo Fisher, Waltham, MA, USA). Cross-sectional micrographs were obtained by fracturing the wet membrane samples after quickly freezing them in liquid nitrogen.

### 2.5. Microalgae Separation Test

Two microalgae cultures were used, namely *Arthrospira platensis* (*Spirulina*, size range 300–500 µm) and *Chlorella vulgaris* (size range 2–10 µm). Both were cultivated in open ponds at the Microalgae Biorefinery, Universitas Gadjah Mada. *C. vulgaris* was used for comparison as the cell size is significantly smaller than that of *Spirulina*. The microalgae separation experiment was conducted using a dead-end cell filtration test at a fixed pressure of 0.5 bar. To determine the concentration of the permeated microalgae solution, an N4S UV–visible spectrophotometer (Ningbo Hinotek Instrument Co., Ltd., Ningbo, China) was used. The spectrophotometer was operated at a wavelength of 760 nm for *Spirulina platensis* [20] and at a wavelength of 240 nm for *Chlorella vulgaris* [21]. The rejection of solute (SR) was determined by

(3) $$\%SR=\left[1-\frac{C_{p}}{C_{f}}\right]\times 100$$

where $C_{p}$ and $C_{f}$ are the permeated and feed solutions of microalgae concentration, respectively.

## 3. Results and Discussion

### 3.1. Membrane Morphology

**Figure 3:**
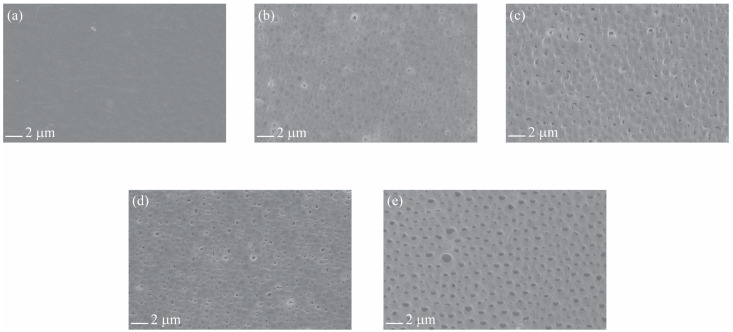
SEM micrographs on top surfaces of 20 wt.% EPS/PVP waste membranes: (**a**) pristine EPS, (**b**) EPS/PVP-2, (**c**) EPS/PVP-4, (**d**) EPS/PVP-6, and (**e**) EPS/PVP-8.

The SEM images of the top surface of the EPS/PVP waste membranes are presented in Figure 3. The incorporation of PVP into the EPS waste dope solution led to noticeable changes in the top surface of the membranes. The pristine EPS waste membrane has a smooth surface; however, the EPS/PVP-2 membrane exhibited subtle alterations in surface topography, characterized by the emergence of small circles protruding downward compared to the pristine EPS waste membrane. As the PVP concentration increased, both the size and quantity of these protrusions increased. The EPS/PVP-8 exhibited nearly uniform surface pores with an estimated maximum diameter of 1 micrometer. These observations align with previous research indicating that the surface pore size of the membrane gradually increases with higher PVP content [18]. Pakan et al. also used PVP as a significant pore-forming agent for fabricating a PVDF-PVP/CuO composite membrane [21]. PVP, as a water-soluble polymer, has the tendency to aggregate around water molecules drawn into the casting film during the inversion phase. This aggregation yields a PVP-rich phase within the membrane [22,23]. According to Strathmann [24], this PVP-rich phase may serve as weak spots in the top layer that penetrates rapidly downward, further forming macrovoids and pressing some pores into a drop-like pore structure.

**Figure 4:**
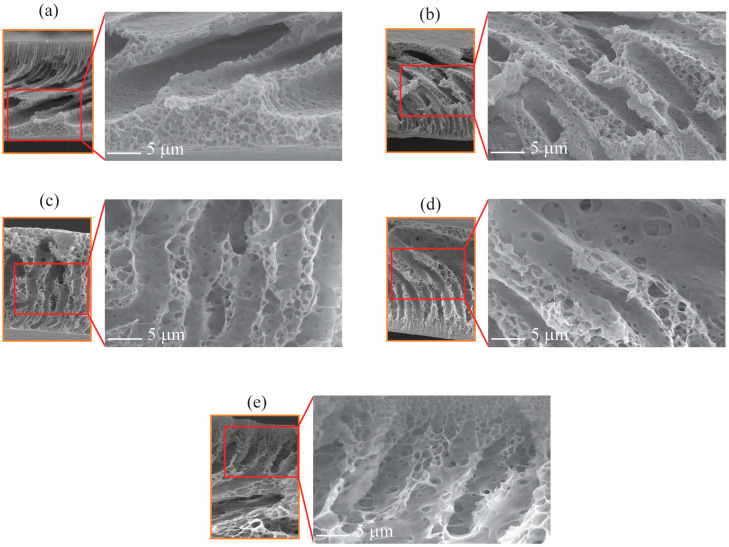
Cross-sectional SEM micrographs of 20 wt.% EPS/PVP waste membranes: (**a**) pristine EPS membrane, (**b**) EPS/PVP-2, (**c**) EPS/PVP-4, (**d**) EPS/PVP-6, and (**e**) EPS/PVP-8.

Figure 4 presents the cross-sectional SEM images of the membrane, observed at 15,000× magnification. Compared to the pristine EPS waste membrane, all EPS/PVP waste membranes exhibit larger pores and fully developed macrovoids, with reduced top layer density. This could be attributed to the water solubility of PVP during phase inversion, which forces the formation of a loose top layer and larger macrovoids. In this work, EPS/PVP-8 exhibited the most open, interconnected finger-like pores supported by the most open pores on the surface. According to Han and Nam [25], macrovoids can be initiated by non-homogeneous demixing on the cast solution surface, and a low concentration of PVP as an additive induces the enhancement of demixing, which explains the enlargement of macrovoids.

### 3.2. Microalgae Harvesting Performance

**Figure 5:**
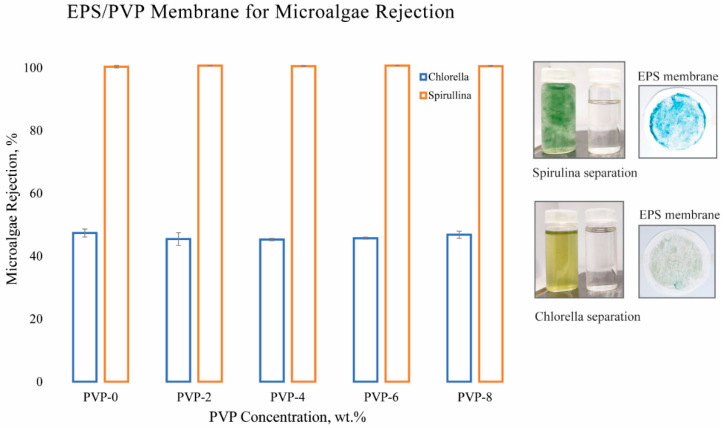
Microalgae harvesting using WEPS membranes with varied PVP concentrations.

A microalgae rejection test was performed to measure the harvesting performance of the EPS waste membrane. To do so, flat sheet EPS waste membranes were randomly sampled from four distinct locations. These membranes were then utilized in the process of microalgae dewatering to extract their biomass. The supernatant was examined to quantify its rejection using a UV–visible spectrophotometer. Figure 5 visually depicts the rejection rate, or harvesting efficiency, of microalgae when interacting with the fabricated EPS waste membranes. Observing the results, both the pristine EPS waste membrane and the ones with added PVP exhibited almost complete biomass extraction of *Spirulina*, with a minimal standard deviation. This finding implies that water reuse is a viable option for subsequent batches of *Spirulina* cultivation when using EPS waste membranes. Sustainable farming is achieved by conserving water, reducing nutrient requirements, as well as helping cleanse the water in case of discharge. In this work, *Chlorella vulgaris,* with a size range of 2–10 µm, was used as a rejection comparison to *Spirulina,* sized 300–500 µm. The *C. vulgaris* supernatant had a pale, greenish color, and it was revealed that the harvesting efficiency was about 50%. The observed color and rejection rate of *C. vulgaris* indicates a likelihood of pore clogging. In microalgae harvesting with the membrane filtration technique, the membrane pore frequently clogged due to the adsorption of extracellular organic matter. These macromolecules, including polysaccharides and fibrous proteins, contribute to irreversible fouling of the membrane, ultimately reducing the efficiency of microalgae harvesting [26].

Harvesting and water reuse pose critical challenges in large-scale microalgae cultivation. Bhave et al. [27] found that microfiltration offers effective separation comparable to ultrafiltration membranes, while operating at lower pressures. Dizge et al. [28] corroborated this finding. Their research demonstrated that membranes with 0.45 µm pore sizes made of mixed cellulose ester (MCE) exhibited higher fluxes compared to membranes with 0.1–0.2 µm pore sizes. In this work, aside from near complete cell recovery of *Spirulina*, a clear supernatant was also observed which is suitable for water reuse. Consequently, larger pore sizes like that observed in EPS waste membranes hold significant potential for microalgae harvesting with significant cost savings and a reduced carbon footprint.

### 3.3. Pure Water Flux Test Experiments

**Figure 6:**
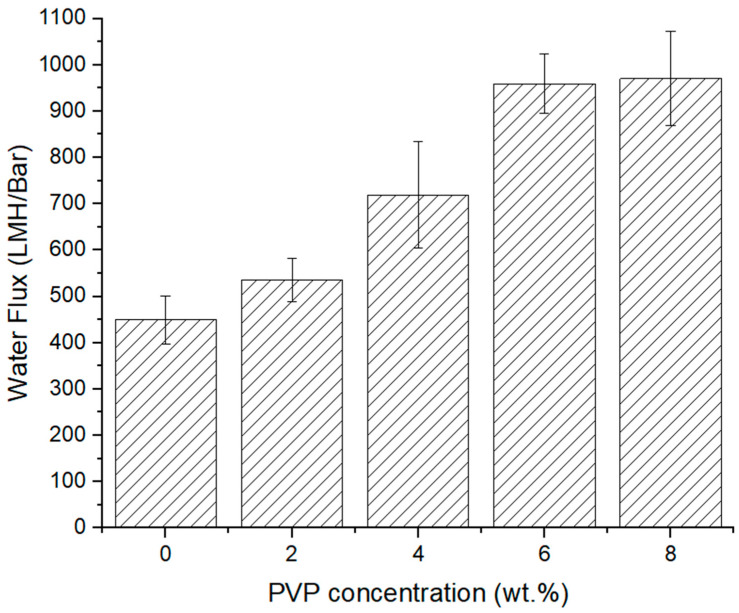
Pure water flux of EPS waste membranes with varied PVP concentrations.

Figure 6 displays the water flux of fabricated EPS waste membranes with varying concentrations of PVP. The water flux is lowest for the pristine EPS waste membrane (449.7 LMH/Bar) and increases with increasing PVP concentration to double fold at PVP-8 (970.5 LMH/Bar), which suggests that the pore size of the pristine EPS waste membrane is the smallest amongst all the membranes. Pure water fluxes increase gradually with increasing PVP content, which is probably due to the increase in surface pore size and macrovoids. The surface SEM micrographs in Figure 3 and Figure 4 clarifies this suggestion. All membranes with PVP have larger open pores, with size and quantity increasing with PVP concentration. It can be seen that all membranes with PVP have larger wall pores, hence better water flow. According to Han and Nam [24], the addition of low-concentration PVP to a cast solution results in the enlargement of macrovoid structures and induced change in surface porosity, which then leads to improved membrane permeate flux. This alteration is attributed to PVP’s ability to enhance phase separation during demixing. Nouzaki et al. [29] observed that their polysulfone membrane became porous when using a casting solution containing PVP as an additive. From this work, they concluded that water-soluble PVP created islands within the membrane during formation, leading to an increase in pore size.

### 3.4. Water Contact Angle Analysis

**Figure 7:**
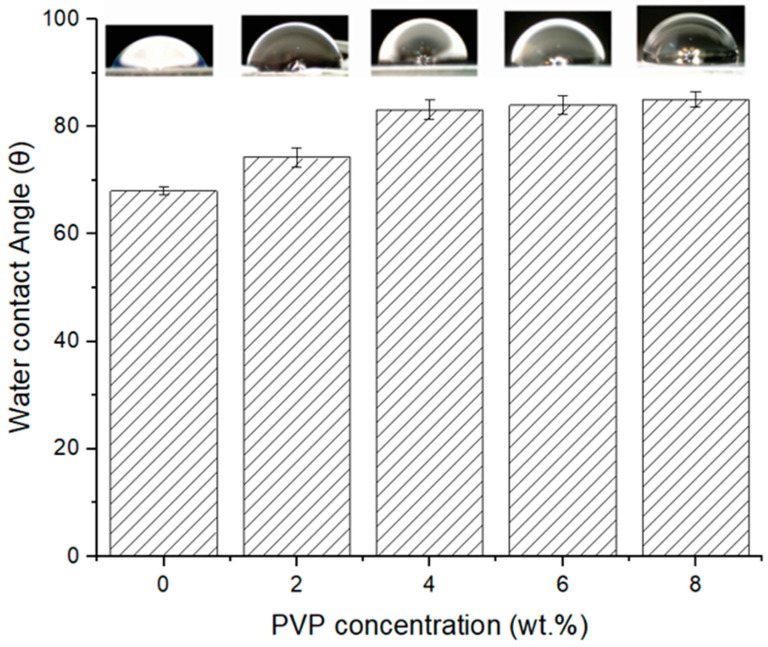
Water contact angle of EPS waste membranes with varied PVP concentrations.

Figure 7 illustrates the water contact angle measurements for the EPS waste membranes. Notably, all the measured angles were below 90°, indicating that all the membranes are hydrophilic. The initial contact angle (CA) of the pristine EPS waste membrane was 68°. The addition of PVP made the contact angle gradually increase to 85°. Because of its water solubility, a significant portion of PVP was washed away from the membrane during the phase inversion process. The reduction of PVP content went against the expected improvement in membrane hydrophilicity. Consequently, the hydrophobicity of the membrane increased as the PVP concentration rose [17].

This finding is aligned with the research of Ghaly et al. [10]. In their research, they fabricated High-Impact Polystyrene (HIPS) waste membranes and revealed that these membranes exhibited either hydrophilic or semi-hydrophilic behavior with contact angles ranging from 69° to 84°. On the contrary, commercial polystyrene (PS) membranes are typically hydrophobic. The observed difference could be attributed to a lower proportion of polystyrene in either EPS or HIPS and the inclusion of additives to make PS products. The presence of impurities likely induced the alteration in membrane surface morphologies, resulting in an increase in hydrophobicity. Given its hydrophilic nature, the membrane is well-suited for water-related applications such as microalgae harvesting or wastewater treatment.

### 3.5. Comparison with Other EPS Waste Membrane Materials

There are few reports in the literature in which EPS waste was used as a separation medium, as presented in Table 1.

Most of the work was conducted using the electrospinning technique, primarily for air filtration media. The majority of membrane fabrication techniques involve electrospinning with various modifications to enhance the efficiency of the air filter media. Shin et al. [5] incorporated micro-glass fibers into an EPS waste dope solution, reporting that this addition improved the separation efficiency of the filter media while also increasing the pressure drop. Munir et al. [12], without adding additives, modified the electrospinning process to be needleless with several inputs. The combination of large and small fiber diameters produced through electrospinning could result in an air filter with an efficiency of up to 99.4%. Ghaly et al. [12] used NIPS to create a flat sheet membrane from EPS waste; however, there is limited information on the membrane’s effectiveness in liquid separation processes. This study, however, utilizes EPS waste for water filtration purposes, specifically for microalgae harvesting. PVP was used to enhance pure water flux. The experimental results indicate a promising future for the upcycled EPS waste membrane in water treatment facilities.

## 4. Conclusions

In this study, Expanded Polystyrene (EPS) waste membranes were prepared using a wet phase inversion procedure and modified by varying the concentration of PVP. The EPS waste, collected from used food packaging, was upcycled into flat sheet membranes through this technique. The overall permeability, morphology, and efficacy in filtering microalgae were investigated. The following conclusions were drawn from this study:

- Introducing PVP into the EPS waste dope solution significantly increased surface pore size and improved connectivity between wall pores, thereby enhancing water flux, as revealed by SEM micrographs.
- These membranes were used to harvest microalgae, resulting in nearly complete biomass recovery for *Spirulina platensis* and 50% recovery for *Chlorella vulgaris*. The EPS/PVP-8 membrane achieved the highest water flux of 970.5 LMH/Bar, which is double that of the pristine EPS waste membrane.
- Despite the increase in hydrophobicity due to the added PVP concentration, all membranes remained hydrophilic. This characteristic makes the upcycled membrane suitable for water-related filtration tasks, including microalgae harvesting.

The supernatant from microalgae separation using the EPS waste membrane can be reused in subsequent cultivations, promoting sustainable farming by conserving water and nutrients. Fabricating membranes from EPS waste offers a promising solution for addressing plastic and water pollution in the future.

## Figures and Tables

**Table 1:** Comparison of upcycled EPS membranes from other works.

EPS wt.%	Additives	Solvent	Technique	Membrane Type	PWF	WCA	Purpose	Ref.
15%	PTFE + SiO_2_	DMF + C_3_H_6_O	Core-shell electrospinning	Microfiber	-	172.4°	Anti-fogging, debris removal	[8]
20%	Micro glass fiber	DMAc	Electrospinning	Nanofiber	-	-	Water-in-oil separation	[5]
15%	-	DMAc	Electrospinning	Nanofiber	-	-	Air filter media	[30]
20%	-	DMF	Needleless electrospinning	Nanofiber	-	179.7	Air filter for facemask	[12]
15%	-	DMF	Electrospinning	Nanofiber	-	153	Air filter media	[31]
18%	-	DMF	NIPS	Flat sheet	-	-	Liquid filtration	[11]
20%	PVP	NMP	NIPS	Flat sheet	970.5	85	Microalgae harvesting	This work

## Data Availability

The original contributions presented in the study are included in the article, further inquiries can be directed to the corresponding author.
